# Sentinel Cards Provide Practical SARS-CoV-2 Monitoring in School Settings

**DOI:** 10.1128/msystems.00109-22

**Published:** 2022-06-15

**Authors:** Victor J. Cantú, Karenina Sanders, Pedro Belda-Ferre, Rodolfo A. Salido, Rebecca Tsai, Brett Austin, William Jordan, Menka Asudani, Amanda Walster, Celestine G. Magallanes, Holly Valentine, Araz Manjoonian, Carrissa Wijaya, Vinton Omaleki, Stefan Aigner, Nathan A. Baer, Maryann Betty, Anelizze Castro-Martínez, Willi Cheung, Peter De Hoff, Emily Eisner, Abbas Hakim, Alma L. Lastrella, Elijah S. Lawrence, Toan T. Ngo, Tyler Ostrander, Ashley Plascencia, Shashank Sathe, Elizabeth W. Smoot, Aaron F. Carlin, Gene W. Yeo, Louise C. Laurent, Anna Liza Manlutac, Rebecca Fielding-Miller, Rob Knight

**Affiliations:** a Department of Bioengineering, University of California San Diego, La Jolla, California, USA; b Department of Pediatrics, University of California San Diego, La Jolla, California, USA; c Expedited COVID Identification Environment (EXCITE) Laboratory, Department of Pediatrics, University of California San Diego, La Jolla, California, USA; d San Diego County Public Health Lab, San Diego, California, USA; e Department of Obstetrics, Gynecology, and Reproductive Sciences, University of California San Diego, La Jolla, California, USA; f Sanford Consortium of Regenerative Medicine, University of California San Diego, La Jolla, California, USA; g Herbert Wertheim School of Public Health, University of California San Diego, La Jolla, California, USA; h San Diego State University, San Diego, California, USA; i Department of Cellular and Molecular Medicine, University of California San Diego, La Jolla, California, USA; j Rady Children's Hospital, San Diego, California, USA; k Division of Infectious Diseases and Global Public Health, Department of Medicine, University of California San Diego School of Medicine, La Jolla, California, USA; l Department of Computer Science and Engineering, University of California San Diego, La Jolla, California, USA; m Center for Microbiome Innovation, Jacobs School of Engineering, University of California San Diego, La Jolla, California, USA; Princeton University

**Keywords:** COVID, environmental sampling, public health, SARS-CoV-2, viral persistence, qPCR

## Abstract

A promising approach to help students safely return to in person learning is through the application of sentinel cards for accurate high resolution environmental monitoring of SARS-CoV-2 traces indoors. Because SARS-CoV-2 RNA can persist for up to a week on several indoor surface materials, there is a need for increased temporal resolution to determine whether consecutive surface positives arise from new infection events or continue to report past events. Cleaning sentinel cards after sampling would provide the needed resolution but might interfere with assay performance. We tested the effect of three cleaning solutions (BZK wipes, Wet Wipes, RNase Away) at three different viral loads: “high” (4 × 10^4^ GE/mL), “medium” (1 × 10^4^ GE/mL), and “low” (2.5 × 10^3^ GE/mL). RNase Away, chosen as a positive control, was the most effective cleaning solution on all three viral loads. Wet Wipes were found to be more effective than BZK wipes in the medium viral load condition. The low viral load condition was easily reset with all three cleaning solutions. These findings will enable temporal SARS-CoV-2 monitoring in indoor environments where transmission risk of the virus is high and the need to avoid individual-level sampling for privacy or compliance reasons exists.

**IMPORTANCE** Because SARS-CoV-2, the virus that causes COVID-19, persists on surfaces, testing swabs taken from surfaces is useful as a monitoring tool. This approach is especially valuable in school settings, where there are cost and privacy concerns that are eliminated by taking a single sample from a classroom. However, the virus persists for days to weeks on surface samples, so it is impossible to tell whether positive detection events on consecutive days are a persistent signal or new infectious cases and therefore whether the positive individuals have been successfully removed from the classroom. We compare several methods for cleaning “sentinel cards” to show that this approach can be used to identify new SARS-CoV-2 signals day to day. The results are important for determining how to monitor classrooms and other indoor environments for SARS-CoV-2 virus.

## OBSERVATION

For the last 2 years, the SARS-CoV-2 pandemic has disrupted lives and caused millions of deaths globally. Due to the high risk of virus transmission in indoor settings, schools have been forced to convert to remote learning (1). Although remote learning can be convenient for some, not every child has access to a stable Internet connection and a supportive, quiet learning environment (2, 3). Therefore, most child health authorities are recommending a return to in-person learning, if it can be conducted safely (4). Effective SARS-CoV-2 monitoring is crucial to allow for in-person learning to resume safely and widely (5), with the goal of restoring education equity. However, performing daily nasal swabs to monitor the spread of the disease has high financial and labor costs, and often runs into difficulties with consent and reporting of results to relevant public health authorities.

Wastewater and environmental monitoring strategies have been developed (6–8) and implemented (9) as a means of circumventing clinical swabs. We have already demonstrated that viral signals from COVID-19 patients in indoor environments commonly accumulate on high-touch surfaces and the floors in front of features with high interaction times (8). Additionally, SARS-CoV-2 RNA has been demonstrated to persist for up to a week on several indoor surface materials (7, 10), making it difficult to understand exactly when an infected individual came into contact with a surface or if consecutive positives are from new deposition events. Thus, an effective postsampling cleaning procedure needs to be established in order to increase temporal resolution and ensure that consecutive positives are from new infection events.

To increase the temporal resolution of proven environmental pipelines (9, 11), we tested resetting SARS-CoV-2 RNA signal with a mock sentinel surface. Here, a sentinel surface is a surface used as an environmental monitoring tool for detecting whether or not an infected individual was recently present in an indoor space. The mock sentinel surfaces we used were 100 cm^2^ laminated cards. The sentinel cards were inoculated with 10 μL of a dilution series of heat-inactivated SARS-CoV-2 particles (strain WA-1, SA-WA1/2020) in water and then wiped with a cleaning solution each day for 5 days. Samples were collected by swabbing the sentinel cards preinoculation, postinoculation, and post wipe (Fig. S1).

10.1128/msystems.00109-22.1 FIG S1 Diagram of sampling events for each day of the experiment. Each day the sentinel cards were swabbed pre- and postinoculation and post wiping. Download FIG S1, PDF file, 0.2 MB. Copyright © 2022 Cantú et al. 2022 Cantú et al. https://creativecommons.org/licenses/by/4.0/ This content is distributed under the terms of the Creative Commons Attribution 4.0 International license.

For this study we used three viral loads: “high” (4 × 10^4^ GE/mL), “medium” (1 × 10^4^ GE/mL), and “low” (2.5 × 10^3^ GE/mL) dilutions of SARS-CoV-2 viral genomic equivalents, as measured by droplet digital PCR. These concentrations were chosen to bracket the ranges we typically observed in classrooms during Safer At School Early Alert (SASEA) (9). We used two different transport media: SDS (0.5% wt/vol sodium dodecyl sulfate, Acros Organics, 230420025), which we have previously shown to yield superior results in SARS-CoV-2 molecular assays (11), and VTM (Viral Transport Medium, NEST Scientific USA, 202016), which the FDA has approved for transporting SARS-CoV-2 samples that will be used for molecular or antigen testing (12). The recipe for VTM as recommended by the CDC is Hanks’ Balanced Salt Solution with 2% fetal bovine serum (FBS), 100 μg/mL gentamicin, and 0.5 μg/mL amphotericin B (13). We chose to use SDS at a concentration of 0.5% because it has already been shown to effectively inactivate SARS-CoV-2 after 30 min of contact time (14, 15). However, local public health laboratories performing SARS-CoV-2 monitoring on a larger scale, such as the San Diego County Public Health Laboratory, currently employ VTM for sample collection.

We tested three cleaning methods: benzalkonium chloride (BZK) antiseptic towelettes (Dynarex, 1331), Wet Wipe towelettes (Royal, RF1MB), and paper towels moistened with RNase Away (ThermoFisher Scientific, 10328011). BZK wipes contain 0.13% benzalkonium chloride, which is an antiseptic and a quaternary ammonium compound; Wet Wipes contain 1% bleach (sodium hypochlorite). Both BZK and sodium hypochlorite are effective at inactivating SARS-CoV-2, as well as other viruses, at relevant concentrations (16–19), and are feasible to implement in school settings due to their cost and packaging (prepackaged wipes are easy to distribute). RNase Away is a dilution of sodium hydroxide and was included because it is recommended by the FDA to minimize nucleic acid contamination (20, 21). However, it only serves as a positive control, since it would not be practical for use in schools.

To continue benchmarking proven environmental pipelines (7, 9, 11) and to account for potential interactions, we used a factorial study design covering two swabbing media (SDS, VTM), three cleaning solutions (BZK wipes, Wet Wipes, RNase Away), and three viral spike-in concentrations (High, Medium, Low). Each condition was performed in triplicate for a total of 54 cards. A three-step swabbing process was performed on each card over a 5-day period (Fig. S1). First, we swabbed each card at the start of the day (Step 1). Next, the viral spike-in was added to the card and a second swab was collected (Step 2). Lastly, the card was wiped with the cleaning solution and a final swab was collected (Step 3). Extraction and RT-qPCR were performed as described in our previous work, with VTM samples processed by the Perkin Elmer pipeline and SDS samples processed by the Thermo Scientific pipeline described in that work (11).

**FIG 1:**
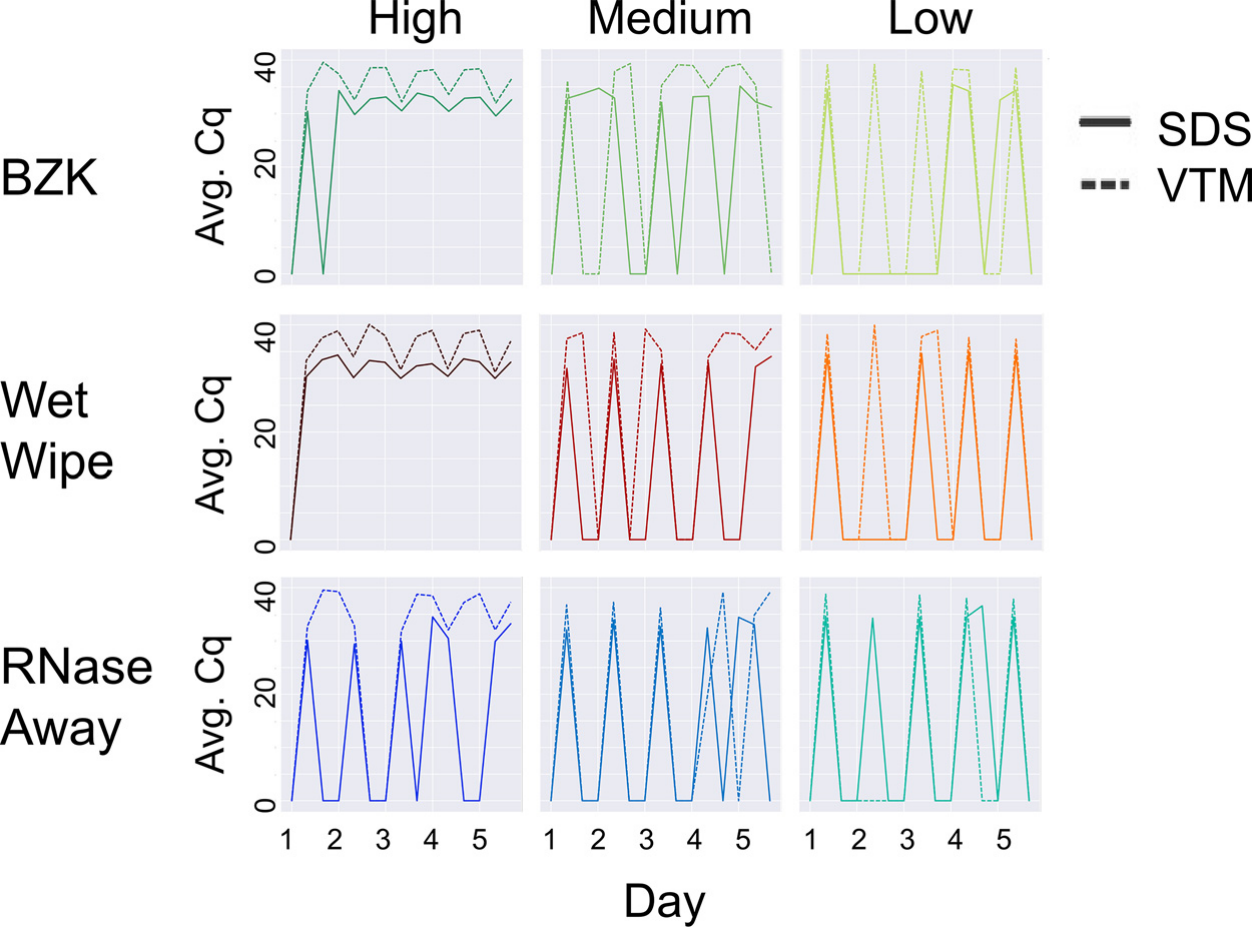
Effect of cleaning solution at high, medium, and low viral load with different swabbing media. On each day, three samples were taken (i): before addition of viral particles (ii), after addition, and (iii) after cleaning. Therefore, the expected pattern is a train of 5 spikes, starting at zero, rising to the maximum Cq value, returning to zero the same day, and staying at zero until the next day, as seen for SDS in the low load condition with RNase Away (bottom right panel, solid lines). High, medium, and low viral load were defined as (4 × 10^4^ GE/mL), (1 × 10^4^ GE/mL), and (2.5 × 10^3^ GE/mL), respectively. Average Cq (Avg. Cq) was calculated as a mean Cq value from three samples. Two viral transport media were tested: SDS (0.5% wt/vol sodium dodecyl sulfate and VTM (Viral Transport Medium)). Effective cleaning reset Cq for each day. RNase Away was shown to be effective at each viral load, whereas benzalkonium chloride (BZK) and Wet Wipes were only effective at medium and low viral load.

Our results demonstrated that all of the cleaning methods worked well at low viral load over 5 cleaning cycles, although cleaning failures were more frequent with BZK (Fig. 1). Both Wet Wipes and BZK performed well with SDS at medium viral loads, but only Wet Wipes performed well with VTM under these conditions. As expected from our past work (11), SDS returned lower Cq values (better signal) than VTM on the same samples. Repeat cleaning did not degrade the sentinel card surface or the ability to detect signal.

The difference in performance between the SDS and VTM conditions in terms of resetting signal can be explained by the properties of the swabbing media themselves. SDS is a surfactant meaning it has both a hydrophilic end, which is attracted to water, and a hydrophobic end that is attracted to the lipids making up the SARS-CoV-2 membrane. This allows SDS to disrupt the lipid membrane while at the same time making biomolecules more readily removable from the cards (22). In contrast, the organic matter from the FBS in VTM introduces organic materials which act as viral clumping protective factors and can affect the efficacy of disinfecting agents with regard to inactivating viruses (18). We indeed noticed that swabbing with VTM resulted in a leftover residue on the cards that was more difficult to wipe off regardless of the cleaning solution. The process of cleaning the card is reliant on removing organic material impurities and the BZK wipes are less efficient at this than the Wet Wipes. This poor performance of BZK with dried virus can be understood from a thermodynamic perspective. BZK is a cationic surfactant (positively charged) and will be attracted to the negatively charged virus and card surface. This means that BZK cannot effectively disperse the virus and so has relatively poor performance as a detergent (22). When the swabbing medium is VTM instead of SDS, this effect is amplified which is supported by a study aimed at understanding the result of adding 5% FBS to viral suspensions. FBS showed no influence on the virucidal capabilities of quaternary ammonium compounds *except* for BZK, decreasing the efficacy of BZK (23). A second study provides possible further insight for the difference in performance between BZK and sodium hypochlorite. In this study, researchers examined the virucidal efficacy of BZK and sodium hypochlorite as measured by cell culture and the genomic integrity of viruses after exposure to the two chemicals measured by RT-PCR. The study found that both compounds effectively inactivated SARS-CoV but that viral RNA could still be detected by PCR when BZK was used (19).

At high viral loads, only the combination of RNase Away and SDS was able to remove the signal. This was as expected since RNase Away is often used to ensure environments are RNA free for sensitive molecular assays (24, 25). Because the combination of RNase Away and SDS cannot be used at scale with existing infrastructure, we recommend sentinel card replacement at earliest convenience, rather than cleaning, if high viral loads (Cq < 30 with SDS or Cq < 35 with VTM) are detected on a sentinel card.

**FIG 2:**
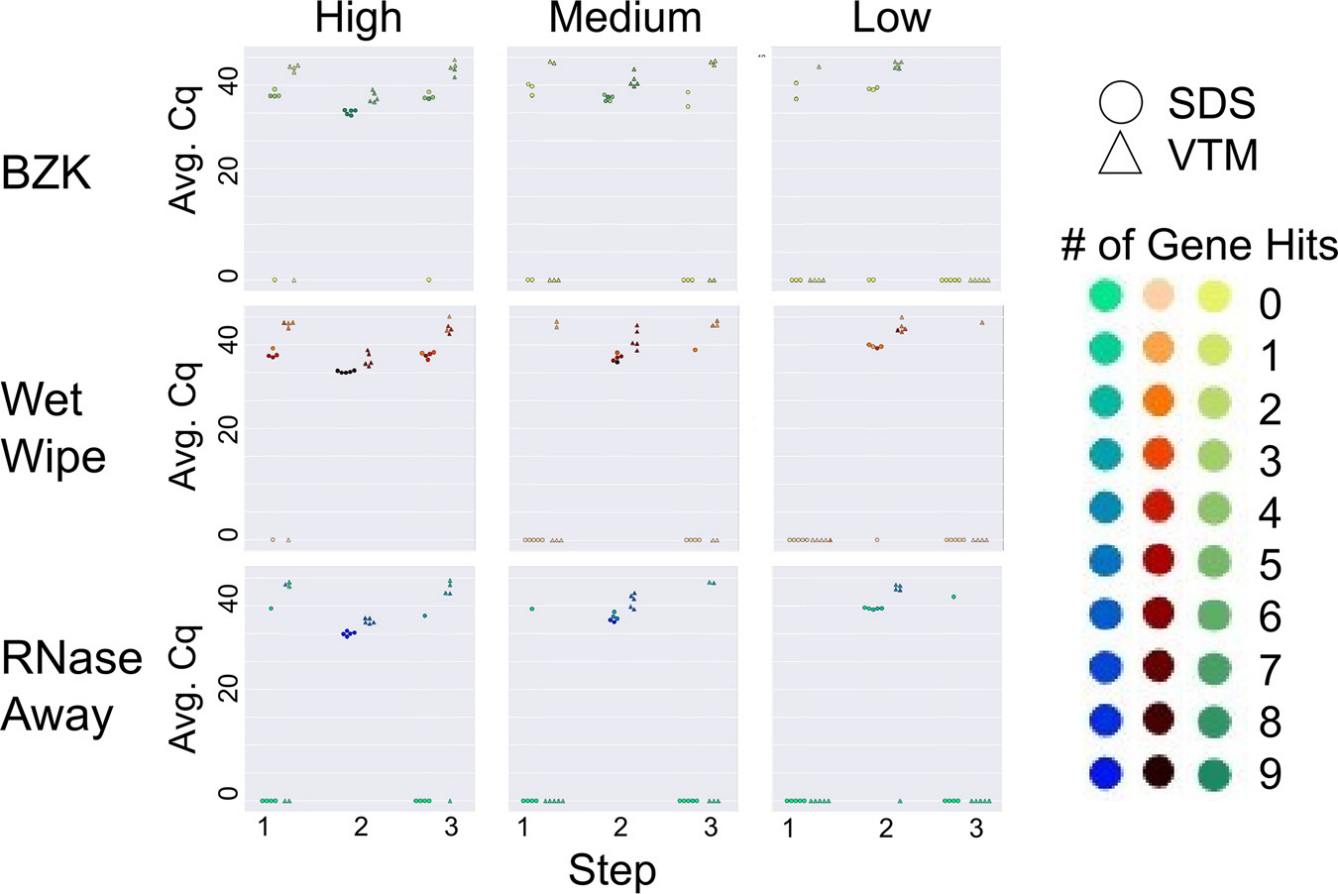
Cleaning solution efficiency after deliberate addition of viral load. Sampling was performed in three steps: initial virus amount (blank) was sampled from the wall for Step 1. Virus was deliberately loaded on the surface and sampled for Step 2. The surface was cleaned with different cleaning methods and sampled for qPCR analysis for Step 3. High, medium, and low viral load were defined as (4 × 10^4^ GE/mL), (1 × 10^4^ GE/mL), and (2.5 × 10^3^ GE/mL), respectively. Average Cq (Avg. Cq) was calculated as a mean Cq value from three samples. Two viral transport media were tested: SDS (0.5% wt/vol sodium dodecyl sulfate and VTM (Viral Transport Medium)). Effective cleaning reset Cq for each day (steps 1 and 3), whereas ineffective cleaning retained high viral load (nonzero Cq) at these steps. The number of gene hits refers to how many gene targets were amplified during RT-qPCR across the triplicate samples: the qPCR method for the SDS samples targeted 3 genes for a total of 9 possible genes amplified while the method for the VTM samples targeted 2 genes for a total of 6 possible gene hits.

An important consideration is the number of distinct genes recovered as matching in the RT-qPCR process, as this can make the difference between a sample being called as SARS-CoV-2 positive versus invalid. Because the peaks with the same viral load applied were highly reproducible across multiple days (reaching the same height in Fig. 1), for this analysis we could treat each day as a replicate of the preapplication, postapplication, and postcleaning sample conditions that were collected on each day. Fig. 2 shows the reproducibility of replicates with cleaning, including the number of genes amplified. Under low load conditions, as expected, cleaning was effective and nonzero values occurred nearly always postapplication and disappeared on cleaning, with the exception of VTM samples which sometimes carried over (right hand column in Fig. 2). In contrast, in the high load condition (left hand column in Fig. 2), cleaning was nearly always ineffective except with RNase Away, not practical for classroom use. In the medium condition (middle column), all cleaning methods were effective with SDS, but none were effective with VTM – the slightly higher cluster of Cq values are obtained with VTM in each case, consistent with expectations and with Fig. 1.

Taken together, these results indicate that sentinel cards are an effective and practical solution for SARS-CoV-2 classroom monitoring, but that they must be cleaned carefully in order to remove carryover signal, and this process is easier with samples collected in SDS than in VTM (although cleaning with VTM is still possible). Because removing high viral load from sentinel cards is challenging, strong positives should be removed rather than cleaned. These findings are an important step to deployment of these cards at scale in projects such as SASEA.

## References

[1] Black E, Ferdig R, Thompson LA. 2021. K-12 virtual schooling, COVID-19, and student success. JAMA Pediatr 175:119–120. doi:10.1001/jamapediatrics.2020.3800. 32780093

[2] van Lancker W, Parolin Z. 2020. COVID-19, school closures, and child poverty: a social crisis in the making. Lancet Public Health 5:e243–e244. doi:10.1016/S2468-2667(20)30084-0. 32275858 PMC7141480

[3] White A, Liburd LC, Coronado F. 2021. Addressing racial and ethnic disparities in COVID-19 among school-aged children: are we doing enough? Prev Chronic Dis 18:1–11. doi:10.5888/pcd18.210084. PMC8220967 34081577

[4] Rubin D, Coffin S, Fisher B, Gerber J, Matone M. 2022. Guidance for In-person Education in K-12 Educational Settings. Children’s Hospital of Philadelphia.

[5] Johnson KE, Stoddard M, Nolan RP, White DE, Hochberg NS, Chakravarty A. 2021. In the long shadow of our best intentions: model-based assessment of the consequences of school reopening during the COVID-19 pandemic. PLoS One 16:e0248509. doi:10.1371/journal.pone.0248509. 33765026 PMC7993767

[6] Karthikeyan S, Nguyen A, McDonald D, Zong Y, Ronquillo N, Ren J, Zou J, Farmer S, Humphrey G, Henderson D, Javidi T, Messer K, Anderson C, Schooley R, Martin NK, Knight R. 2021. Rapid, Large-scale wastewater surveillance and automated reporting system enable early detection of nearly 85% of COVID-19 Cases on a university campus. mSystems 6. doi:10.1128/mSystems.00793-21. PMC8409724 34374562

[7] Salido RA, Cantú VJ, Clark AE, Leibel SL, Foroughishafiei A, Saha A, Hakim A, Nouri A, Lastrella AL, Castro-Martínez A, Plascencia A, Kapadia BK, Xia B, Ruiz CA, Marotz CA, Maunder D, Lawrence ES, Smoot EW, Eisner E, Crescini ES, Kohn L, Franco Vargas L, Chacón M, Betty M, Machnicki M, Wu MY, Baer NA, Belda-Ferre P, de Hoff P, Seaver P, Ostrander RT, Tsai R, Sathe S, Aigner S, Morgan SC, Ngo TT, Barber T, Cheung W, Carlin AF, Yeo GW, Laurent LC, Fielding-Miller R, Knight R. 2021. Analysis of SARS-CoV-2 RNA persistence across indoor surface materials reveals best practices for environmental monitoring programs. mSystems 6. doi:10.1128/mSystems.01136-21. PMC8562474 34726486

[8] Cantú VJ, Salido RA, Huang S, Rahman G, Tsai R, Valentine H, Magallanes CG, Aigner S, Baer NA, Barber T, Belda-Ferre P, Betty M, Bryant M, Casa-Maya M, Castro-Martínez Chacón M, Cheung W, Crescini ES, de Hoff P, Eisner E, Farmer S, Hakim A, Kohn L, Lastrella AL, Lawrence ES, Morgan SC, Ngo TT, Nouri A, Plascencia A, Ruiz CA, Sathe S, Seaver P, Shwartz T, Smoot EW, Ostrander T, Valles T, Yeo GW, Laurent LC, Fielding-Miller R, Knight R. 2021. SARS-CoV-2 distribution in residential housing suggests contact deposition and correlates with *Rothia sp*. (accepted for publication). 10.1128/msystems.01411-21 PMC9239251 35575492

[9] Fielding-Miller R, Karthikeyan S, Gaines T, Garfein RS, Salido R, Cantu V, Kohn L, Martin NK, Wijaya C, Flores M, Omaleki V, Majnoonian A, Gonzalez-Zuniga P, Nguyen M, Vo A, Le T, Duong D, Hassani A, Dahl A, Tweeten S, Jepsen K, Henson B, Hakim A, Birmingham A, Mark AM, Nasamran CA, Rosenthal SB, Moshiri N, Fisch KM, Humphrey G, Farmer S, Tubb HM, Valles T, Morris J, Kang J, Khaleghi B, Young C, Akel AD, Eilert S, Eno J, Curewitz K, Laurent LC, Rosing T, Knight R, SEARCH. 2021. Wastewater and surface monitoring to detect COVID-19 in elementary school settings: the Safer at School Early Alert project. medRxiv: the preprint server for health sciences. doi:10.1101/2021.10.19.21265226. PMC9939935 36844610

[10] Renninger N, Nastasi N, Bope A, Cochran SJ, Haines SR, Balasubrahmaniam N, Stuart K, Bivins A, Bibby K, Hull NM, Dannemiller KC. 2021. Indoor dust as a matrix for surveillance of COVID-19. mSystems 6. doi:10.1128/mSystems.01350-20. PMC8547012 33850045

[11] Cantú VJ, Belda-Ferre P, Salido RA, Tsai R, Austin B, Jordan W, Asudani M, Walster A, Magallanes CG, Valentine H, Manjoonian A, Wijaya C, Omaleki V, Sanders K, Aigner S, Baer NA, Betty M, Castro-Martínez A, Chacón M, Cheung W, Crescini ES, de Hoff P, Eisner E, Hakim A, Kapadia B, Lastrella AL, Lawrence ES, Ngo TT, Ostrander T, Sathe S, Seaver P, Smoot EW, Carlin AF, Yeo GW, Laurent LC, Manlutac AL, Fielding-Miller R, Knight R. 2022. Implementation of practical surface SARS-CoV-2 surveillance in school settings (provisionally accepted for publication). 10.1128/msystems.00103-22 PMC9426517 35703437

[12] U.S. Department of Health and Human Services Food and Drug Administration. 2021. Enforcement policy for viral transport media during the coronavirus disease 2019 (COVID-19) public health emergency (revised). Available at: https://www.fda.gov/regulatory-information/search-fda-guidance-documents/enforcement-policy-viral-transport-media-during-coronavirus-disease-2019-covid-19-public-health. (Accessed: 12 March 2022).

[13] Centers for Disease Control and Prevention. Preparation of Viral Transport Medium. 2020. Available at: https://www.cdc.gov/coronavirus/2019-ncov/downloads/Viral-Transport-Medium.pdf. (Accessed: 12 March 2022).

[14] Patterson EI, Prince T, Anderson ER, Casas-Sanchez A, Smith SL, Cansado-Utrilla C, Solomon T, Griffiths MJ, Acosta-Serrano Á, Turtle L, Hughes GL. 2020. Methods of inactivation of SARS-CoV-2 for downstream biological assays. J Infect Dis 222:1462–1467. doi:10.1093/infdis/jiaa507. 32798217 PMC7529010

[15] Welch SR, Davies KA, Buczkowski H, Hettiarachchi N, Green N, Arnold U, Jones M, Hannah MJ, Evans R, Burton C, Burton JE, Guiver M, Cane PA, Woodford N, Bruce CB, Roberts ADG, Killip MJ. 2020. Analysis of inactivation of SARS-CoV-2 by specimen transport media, nucleic acid extraction reagents, detergents, and fixatives. J Clin Microbiol 58. doi:10.1128/JCM.01713-20. PMC7587104 32839250

[16] Ogilvie BH, Solis-Leal A, Lopez JB, Poole BD, Robison RA, Berges BK. 2021. Alcohol-free hand sanitizer and other quaternary ammonium disinfectants quickly and effectively inactivate SARS-CoV-2. J Hosp Infect 108:142–145. doi:10.1016/j.jhin.2020.11.023. 33259880 PMC7700010

[17] Kwok CS, Dashti M, Tafuro J, Nasiri M, Muntean EA, Wong N, Kemp T, Hills G, Mallen CD. 2021. Methods to disinfect and decontaminate SARS-CoV-2: a systematic review of in vitro studies. Ther Adv Infect Dis 8. 10.1177/2049936121998548 PMC7970236 33796289

[18] Lin Q, Lim JYC, Xue K, Yin P, Yew M, Owh C, Chee PL, Loh XJ. 2020. Sanitizing agents for virus inactivation and disinfection. View 1:e16. doi:10.1002/viw2.16. 34766164 PMC7267133

[19] Ansaldi F, Banfi F, Morelli P, Valle L, Durando P, Sticchi L, Contos S, Gasparini R, Crovari P. 2004. SARS-CoV, influenza A and syncitial respiratory virus resistance against common disinfectants and ultraviolet irradiation. J Prev Med Hyg 45:5–8.

[20] U.S. Food and Drug Administration. 2021. CovidNow SARS-CoV-2 assay–letter of authorization. Available at: https://www.fda.gov/media/153172/download. (Accessed: 12 March 2022).

[21] Lighthouse Lab Services. 2021. CovidNow SARS-CoV-2 assay–instructions for use. Available at: https://www.lighthouselabservices.com/wp-content/uploads/2021/10/IFU-CovidNow-20211012-FINAL-CLEAN.pdf. (Accessed: 12 March 2022).

[22] Simon M, Veit M, Osterrieder K, Gradzielski M. 2021. Surfactants–compounds for inactivation of SARS-CoV-2 and other enveloped viruses. Curr Opin Colloid Interface Sci 55:101479. doi:10.1016/j.cocis.2021.101479. PMC8196227 34149296

[23] Tsujimura K, Murase H, Bannai H, Nemoto M, Yamanaka T, Kondo T. 2015. Efficacy of five commercial disinfectants and one anionic surfactant against equine herpesvirus type 1. J Vet Med Sci 77:1545–1548. doi:10.1292/jvms.15-0030. 26074409 PMC4667681

[24] Quigley MF, Almeida JR, Price DA, Douek DC. 2011. Unbiased molecular analysis of T cell receptor expression using template-switch anchored RT-PCR. Curr Protoc Immunol CHAPTER:Unit10.33. 10.1002/0471142735.im1033s94 PMC3154014 21809317

[25] Sessitsch A, Gyamfi S, Stralis-Pavese N, Weilharter A, Pfeifer U. 2002. RNA isolation from soil for bacterial community and functional analysis: evaluation of different extraction and soil conservation protocols. J Microbiol Methods 51:171–179. doi:10.1016/s0167-7012(02)00065-9. 12133609

