# Supplementary figures and images for: Sentinel Cards Provide Practical SARS-CoV-2 Monitoring in School Settings
Source: mSystems. 2022 Jun 15;7(4):e00109-22. doi: 10.1128/msystems.00109-22 (PMC9426498; doi:10.1128/msystems.00109-22)

# Measuring SARS-CoV-2 signal removal

Inoculate

Swab 1

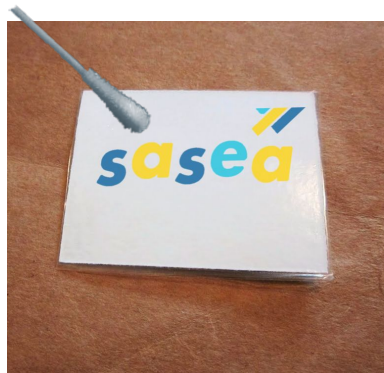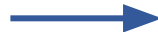

Swab 2

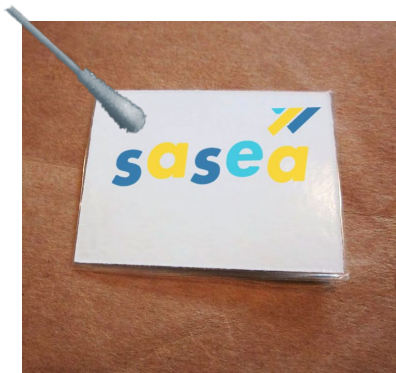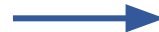

Swab 3

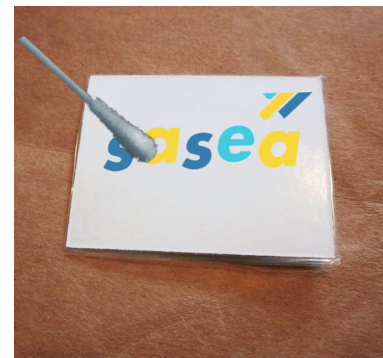

Wipe

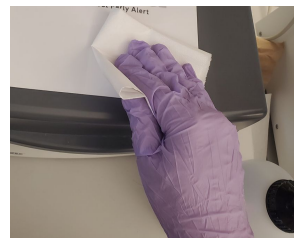

Supplement: FIG S1 [file msystems.00109-22-s0001.pdf]
